# Atomic Force Microscope Guided SERS Spectra Observation for Au@Ag-4MBA@PVP Plasmonic Nanoparticles

**DOI:** 10.3390/molecules24203789

**Published:** 2019-10-21

**Authors:** Liu Yang, Libei Xu, Xiuju Wu, Hui Fang, Shenfei Zhong, Zhuyuan Wang, Jing Bu, Xiaocong Yuan

**Affiliations:** 1 Nanophotonics Research Center, Shenzhen University, Shenzhen 518060, China; yangliu@hisense.com (L.Y.); Xulibei2017@email.szu.edu.cn (L.X.); xcyuan@szu.edu.cn (X.Y.); 2 Institute of Modern Optics, Nankai University, Tianjin 30071, China; 3 Advanced Photonic Center, Southeast University, Nanjing 210096, China; sfzong@seu.edu.cn (S.Z.); wangzy@seu.edu.cn (Z.W.); 4 College of Electronic Science and Technology, Shenzhen University, Shenzhen 518060, China; jingbu@szu.edu.cn

**Keywords:** plasmonic nanoparticles, SERS probes, SERS spectra, AFM imaging, confocal Raman spectrometer, photothermal effect

## Abstract

Recently polymer encapsulated surface-enhanced-Raman-scattering (SERS) probes with internal noble metal core–shell structure has found growing applications in biomedical applications. Here we studied the SERS spectra of Au@Ag–4MBA@PVP (4MBA: 4-mercaptobenzoic acid; PVP: polyvinylpyrrolidone) plasmonic nanoparticles produced from a chemical reduction method. By linking the atomic force microscope (AFM) with the homebuilt confocal Raman spectrometer thus to use AFM images as guidance, we realized the measurement of the SERS spectra from separated nanoparticles. We investigated the cases for single nanoparticles and for dimer structures and report several observed results including SERS spectra linearly scaled with laser power, abrupt boosting and abnormal shape changing of SERS spectra for dimer structures. Based on the finite element method simulation, we explained the observed ratio of SERS signals between the dimer structure and the single nanoparticle, and attributed the observed abnormal spectra to the photothermal effect of these plasmonic nanoparticles. Our study provides valuable guidance for choosing appropriate laser power when applying similar SERS probes to image biological cells.

## 1. Introduction

Plasmonic nanoparticles adsorbed with Raman molecules can work as SERS probes and already show great promise in wide biomedical application, especially for imaging biological cells [1,2]. Compared to fluorescence labels, SERS probes own the advantages of being immune to the photobleaching and being capable of multiplex coding under a single laser excitation. As a common strategy, a thin layer of polymer molecules are applied to encapsulate the nanoparticles to protect the Raman molecules from the biological environment, to help to stabilize the SERS enhancement, and also to control the aggregation of the SERS probes [3].

In this paper, we study the Au@Ag–4MBA@PVP nanoparticles synthesized from a standard procedure of a chemical reduction method. It has been demonstrated that the PVP molecule thin layer not only can protect the Raman reporter (which is the 4MBA molecule here) but also can improve the biocompatibility of such SERS probes and enhance the endocytosis ability with respect to living cells [4]. Au/Ag core–shell nanoparticles are used as the plasmonic template because Ag nanoparticles with sizes bigger than 100 nm are usually difficult to produce and the plasmonic resonance properties of such core–shell nanoparticles can be tuned in a wide range with the flexibility to control the core size and the shell thickness [5]. Here, we report the experiment results of measuring Raman spectra of separated Au@Ag–4MBA@PVP nanoparticles, specifically aiming to compare the difference of single nanoparticles versus dimer structures which previously has not been closely examined. In order to understand the observed SERS signal ratio and some abnormal SERS spectra, we also present and discuss the corresponding simulation results based on the finite element method (FEM). 

We took the approach of atomic force microscopy (AFM) imaging [6] to confirm the morphology of nanoparticles which were simultaneously undergoing Raman spectral measurement. Although TEM imaging can provide much higher resolution, AFM is much easier to couple with the confocal Raman spectrometer [7,8,9] to facilitate the routine simultaneous evaluation. As it is well known, the magnitude of SERS spectra for dimer structures heavily depends on the polarization orientation of the optical beam. In our experiment system, we applied the radially polarized optical beam which is often used in the SERS system [10]. It was found that the dimer axis and the light polarization can be aligned by laterally shifting the nanoparticles relative to the laser focal spot. 

In our experiments, we found some SERS phenomena which were easier to be observed for the dimer structure than the single nanoparticle when the laser power was controlled below a few mW, including the increase of SERS background during the measurement and the Raman signal abrupt change when the laser power was increased. We attributed these to the photothermal effect of SERS plasmonic nanoparticles which recently drew more and more interest [11,12,13,14,15,16]. Although in some cases nanostructures with highly efficient photothermal conversion are pursued such as for solar collectors and energy saving applications [17] or for cancer cell photothermal therapy [18], in this paper we were mainly concerned with the possible photothermal damage effect to the SERS probes. 

## 2. Results

### 2.1. Atom Force Microscope Images and SERS Spectra

Our experiments were performed on the system by linking AFM with the confocal Raman spectrometer. In order to identify the separated nanoparticles, we first looked at the wide-field optical microscopic image and chose an area with some visible scattering spots, and then scanned this area with the AFM tip. By following the suggestion if Reference [6], the AFM scanning was operated under an intermittent contact mode but not the contact mode since the nanoparticles were only weakly adhered to the microslide. To save the scanning time, the AFM image at a relatively large area was first inspected, and then the reduced area encompassing only one nanoparticle or one dimer structure was then scanned with a reduced step interval. 

**Figure 1:**
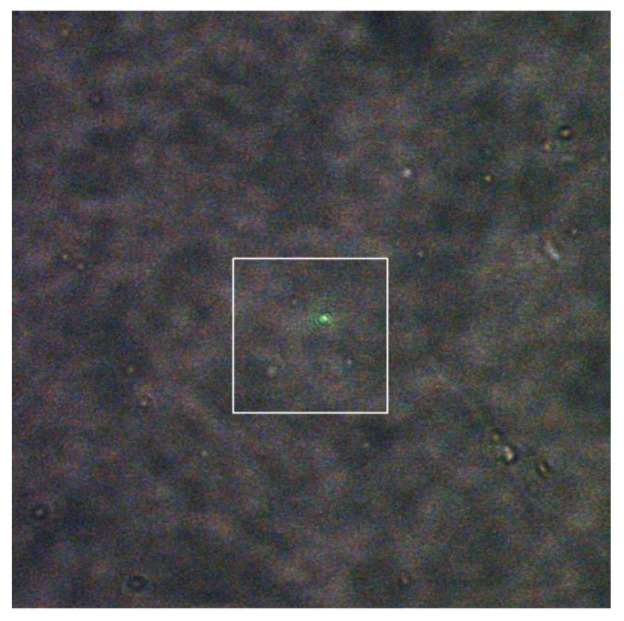
A wide-field optical microscopic image showing bright scattering spots due to nanoparticles. The white square marks the area (9 µm × 9 µm) to obtain the atomic force microscopy (AFM) image as shown in Figure 2a,d, and the greenish light spot shows that the focused laser spot hits on one of the nanoparticles.

**Figure 2:**
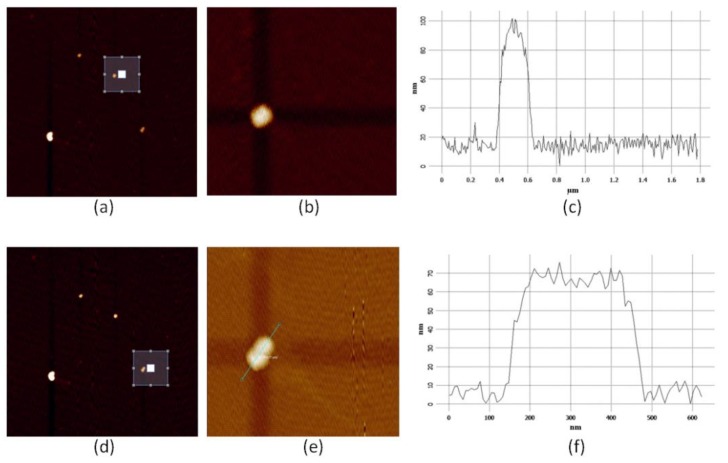
AFM images of two nanoparticle groups in the area as marked in Figure 1. (**a**) AFM image of the whole area of 9 µm × 9 µm where the small white line square indicates the subarea of 1.8 µm × 1.8 µm to be further scanned with higher resolution; (**b**) AFM image of the marked area in (**a**) where a single nanoparticle is enclosed; (**c**) height profile curve taken along the horizontal line in (**b**); (**d**) repeated AFM image for the whole area before scanning another subarea of 1.8 µm × 1.8 µm marked with the small white line square; (**e**) AFM image of the area marked in (**d**) where possibly a dimer structure is enclosed; (**f**) height profile curve taken along the possible dimer axis as marked in (**e**).

Figure 1 and Figure 2 show representative results to illustrate the above procedure, where Figure 1 shows the wide-field optical microscopic image and Figure 2 shows the corresponding AFM images. As can be seen in Figure 1, in the image with the optical field of view of 35 µm × 35 µm, there was a distribution of about 25 bright spots each of which correlated possibly to one single nanoparticle. We then carefully moved the sample by controlling the translation stage to align the laser focal spot to coincide with one of the bright spots until a greenish light scattering spot appeared. Since we already aligned the AFM tip with the laser focal spot, an area enclosing the laser focal spot marked with the white square could be set as the AFM scanning area.

Figure 2a shows the AFM image of the area chosen from the wide-field optical microscopic inspection, where the marked subarea of 1.8 µm × 1.8 µm was further scanned with the resolution of five times higher, resulting in the AFM image shown in Figure 2b (both images are composed by 256 × 256 scanning steps). In Figure 2d, the AFM imaging of the whole area was repeated before choosing another subarea to scan with the higher resolution. As can be seen, same as Figure 2b, Figure 2e also shows more details of the morphology.

To get accurate information about the nanoparticle size, the height profile curve related to the AFM image can be drawn, as shown in Figure 2c and Figure 2f where the curve in Figure 2c was plotted along the horizontal line and the curve in Figure 2f was plotted along the green short line marked in Figure 2e. Both profiles showed similar heights around 75 nm which agreed with the size obtained by TEM imaging. The lateral dimension seen from Figure 2c was about 200 nm and that from Figure 2f was about 325 nm. Based on these height profile curves and the fact that the produced nanoparticles were mostly rounded in shape (see Figure 10), we could tentatively postulate that Figure 2c enclosed a single nanoparticle while Figure 2d enclosed a dimer structure. It was noted that the lateral dimension obtained from Figure 2c,f were slightly larger than the particle heights. There were two factors to account for this: one was that the lateral dimension appeared in profile curves already included the convolution effect of the AFM tip (about 20 nm in diameter), and the other was that the nanoparticles may not have been strictly spherical in shape and it was their flattened side sitting on the microslide.

**Figure 3:**
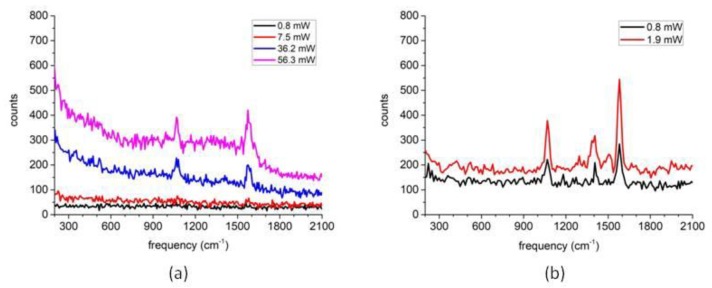
SERS spectra observed from the single nanoparticle and the dimer structure respectively correlating to Figure 2b,e at varying laser powers. (**a**) For the single nanoparticle; (**b**) for the dimer structure. The exposure times for the spectra in (**a**,**b**) were 3 s and 8 s, respectively.

After examining the morphology of the nanoparticles, we then measured their SERS spectra. Figure 3a,b respectively show results for the nanoparticle structures identified in Figure 2b,e. As can be seen, at the laser power range indicated for both cases, the SERS spectra were linearly scaled with the laser powers. It also could be clearly observed that the SERS signal of Figure 3b was much stronger than Figure 3a: taking a close look at the Raman peak at 1580 cm^–1^, we could actually get a ratio of 21 times by normalizing all of the spectra to the respective laser powers and also the exposure times (which were 3 s for the spectra in Figure 3a and 8 s for the spectra in Figure 3b).

Finally, by combining the evaluation for SERS spectra shown in Figure 3 with the examination for the AFM images shown in Figure 2, we could confirm the assignment of the single nanoparticle and the dimer structure previously made merely based on Figure 2. 

### 2.2. Observation of Abnormal SERS Spectra for Dimer Structures

During the experiment, we found that the SERS spectra from single nanoparticles linearly scaled with laser powers could be repeatedly obtained several times when the laser power was below 60 mW, while for the dimer structure the SERS spectra often showed some sudden changes even when the laser power reached only a few mW. 

**Figure 4:**
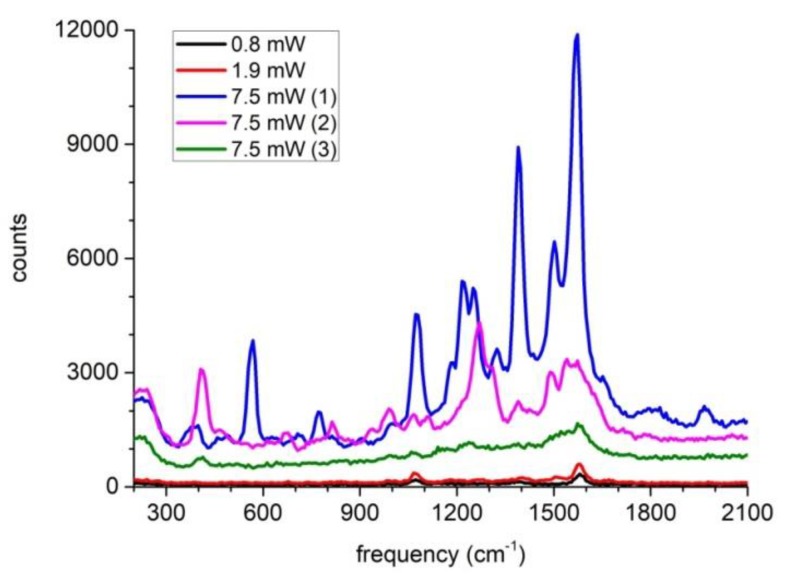
SERS spectra of a dimer structure measured subsequently with different laser power settings.

Figure 4 presents a typical such observation. As can be seen, when the laser power was set in the range from 0.8 mW to 1.9 mW, the SERS spectra varied linearly with the laser power, similar to the observation shown in Figure 3b. However, when the laser power reached 7.5 mW, as shown by the blue curve, the SERS spectrum grew rapidly. The subsequent measurements are shown as the pink curve and the green curve whose SERS signal eventually decreased and at the same time the SERS spectra also changed their shapes. If we compare the Raman peaks among the blue curve, the pink curve and the green curve, we can see the blue curve still characterized the 4MBA molecules (with the 1580 cm^–1^ and 1075 cm^–1^ peaks representing the aromatic ring breathing mode and the 1390 cm^–1^ peak represents the carboxyl stretching mode), while the pink curve already lost the Raman peaks of 4MBA molecules and the green curve showed a broad amorphous-carbon-like Raman peak around 1600 cm^–1^ [19]. 

We tentatively attributed this sudden spectral change due to the photothermal heating of the plasmonic nanoparticles. When the temperature was raised above a threshold value, the PVP layer shrunk and the immediate consequence was that the gap size suddenly decreased thus the SERS signal was boosted significantly as shown by the blue curve. When the temperature was still increasing, the 4MBA molecules were degraded thus the SERS spectrum was dominantly contributed by the PVP molecules as shown by the pink curve. Finally, the PVP molecules were thermally degraded until an amorphous-carbon-like material was formed as shown by the green curve.

**Figure 5:**
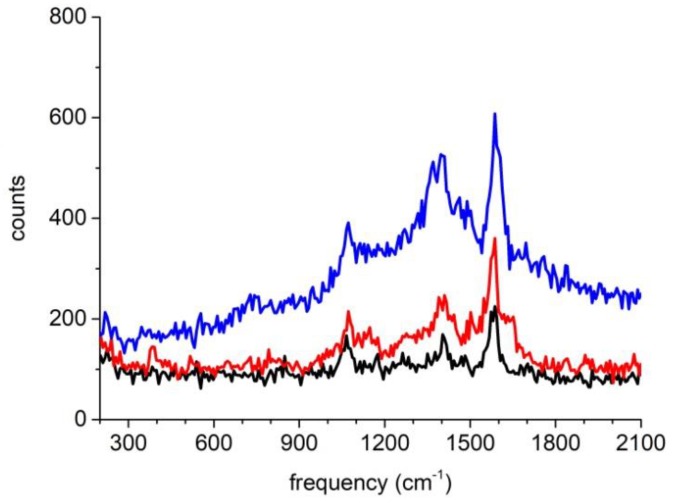
SERS spectra of a dimer structure measured continually at the fixed laser power setting of 0.8 mW (with the time order from black curve, next red curve, then blue curve).

We also found that sometimes even when the laser power was controlled at a low value such as 0.8 mW, for dimer structures an increase of SERS spectral background could be observed as shown in Figure 5. It is worth noting that the mechanism about forming the SERS spectral background was recently under much debate [14], and it is currently understood to be dominated by inelastic light scattering from the electrons of plasmonic nanoparticles [15]. Recently, correction of such background was actually proved to be very important for retrieving the SERS fingerprint [16].

### 2.3. FEM Simulation of SERS Enhancement Factors and Photothermal Temperature Increase

**Figure 6:**
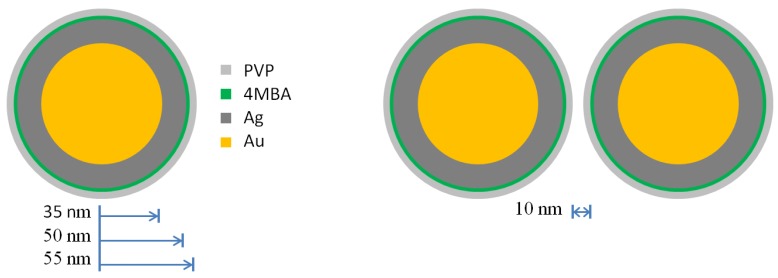
The multilayer sphere structure for modeling a single nanoparticle and a dimer structure: Au core with radius of 35 nm, Ag layer with thickness of 15 nm and PVP layer with thickness of 5 nm, the gap of air with distance of 10 nm.

**Figure 7:**
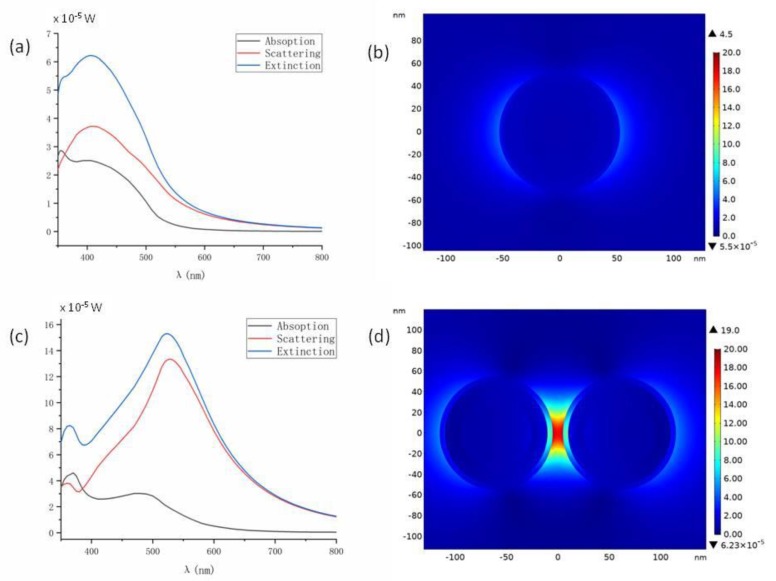
Electromagnetic field finite element method (FEM) stimulation for a single nanoparticle and for a dimer structure. (**a**) Scattering, absorption and extinction spectra for a single nanoparticle; (**b**) electric field enhancement distribution for a single nanoparticle at the resonance wavelength of 406 nm; (**c**) spectra for a dimer structure; (**d**) electric field enhancement distribution for a dimer structure at the resonance wavelength of 530 nm.

**Figure 8:**
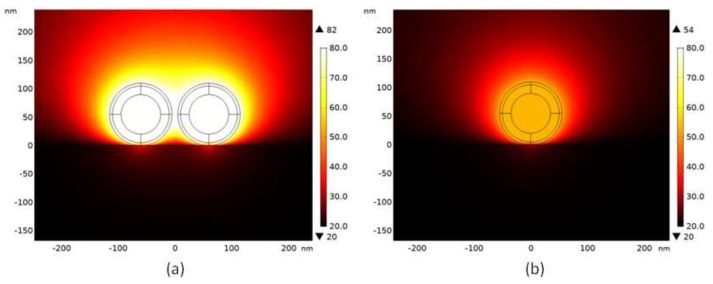
FEM simulation results for the dimer structure (**a**) and for the single nanoparticle (**b**).

In order to understand the different SERS enhancement factor and the different photothermal behavior between the single nanoparticle and the dimer structure, we performed some theoretical calculations based on the FEM simulation. In the simulation, each nanoparticle was modeled by the multilayer sphere structure as shown in Figure 6: an Au core of 35 nm in radius, an Ag layer with thickness of 15 nm, an outer layer of PVP with thickness of 5 nm. For the dimer structure, the was set as 10 nm as can also be visualized in Figure 7d and Figure 8a.

As respectively shown in Figure 7a,c, we first calculated the scattering, absorption and thus the extinction spectra for the single nanoparticle and for the dimer structure. It could be seen that after forming the dimer structure, the plasmonic resonance peaks showed a clear redshift from about 406 nm to 530 nm. Since we set the laser intensity as 1.3 × 10^5^ W/cm^2^ in the simulation, from the spectra we could figure out the corresponding cross sections. For instance, the resonant extinction cross sections in Figure 7a,c are respectively about 5.0 × 10^−10^ and 12 × 10^−10^ cm^2^.

We then simulated the distribution of electrical field enhancement at the respective resonant wavelengths, as shown in Figure 7b,d. As can be seen, for the dimer structure the electrical filed at the gap takes a further enhancement of about 4.2 times. If we consider the fourth power of the electrical field both at 532 cm wavelength, the SERS enhancement at the gap of the dimer structure was as high as 1200 times relative to the SERS enhancement of a single nanoparticle. However, this was the result correlated to the maximum enhancement factor. To simulate the experiment results of the SERS measurement, all of the 4MBA molecules surrounding the nanoparticles needed to be taken account. Therefore, we integrated the SERS enhancement of the whole PVP layer for each nanoparticle where 4MBA molecules were assumed to be uniformly distributed. We obtained the ratio of the integrals of about 60 times (the experiment value for the ratio obtained from Figure 3 was 21 times).

In order to understand how dramatic the temperature can increase due to the photothermal effect, we performed the FEM simulation for both the dimer structure and the single nanoparticle, and the results are shown in Figure 8. As for the simulation settings, the laser intensity was 3.3 × 10^2^ W/cm^2^, the thermal insulation boundary condition was used, and the silicon substrate supporting the nanoparticles was considered. As can be seen, after only 10 ns, the temperature of the dimer structure already increased to 82 °C starting from 20 °C while for the single particles the temperature reached about 54 °C. Therefore, the obtained temperature rise of the dimer structure was much faster. This partly explained the observation that for single nanoparticles the photothermal degradation could only be detected at a much higher laser power above 60 mW (compared to 7.5 mW in the dimer structure case when the photothermal degradation appeared).

## 3. Discussion

With the guidance of AFM imaging, we successfully obtained the Raman spectra of individual multilayer Au@Ag-4MBA@PVP plasmonic nanoparticles. As shown in Figure 3, we observed that at the low laser power setting the dimer nanoparticles and single nanoparticles both own SERS spectra linearly scaled with the laser power. With the help of electromagnetic field FEM simulation as shown in Figure 7, the SERS signal ratio between the dimer structure and the single nanoparticle was calculated to be 60 times which was somewhat larger than the experiment observation of 21 times. We have performed the measurement for five single nanoparticles and five dimer structures, and obtained the SERS signal ratios distributed in the range 5–30 times. We think this was actually reasonable in agreement since the morphology of the nanoparticles could not be strictly defined, and the 4MBA molecules may not have been not uniformly distributed in the PVP layer as assumed in the FEM simulation. 

As shown in Figure 4, we also observed that when the laser power was increased to reach some threshold, the SERS spectra of the dimer nanoparticles often initially showed abrupt boost and later showed the abnormal shapes very different from the SERS spectra of the 4MBA molecules. Based on the heat production FEM simulation as shown in Figure 8, we attributed the phenomena to the photothermal effect of the plasmonic nanoparticles which induced the PVP layer shrinking and the subsequent PVP molecule degradation. 

Our study suggested that when using the Au@Ag-4MBA@PVP nanoparticles to image cells, the laser power should be well controlled to balance the requirement of getting enough SERS signal and the possibility of photothermal degrading. We are aware that to better mimic the situation of labeling SERS probes in cells, the study should be performed for the nanoparticles immersed in liquid. However, the AFM imaging of nanoparticles in aqueous environment is difficult and the effort for such experiments will be put forward in the near future. 

## 4. Materials and Methods

### 4.1. Experimental System

**Figure 9:**
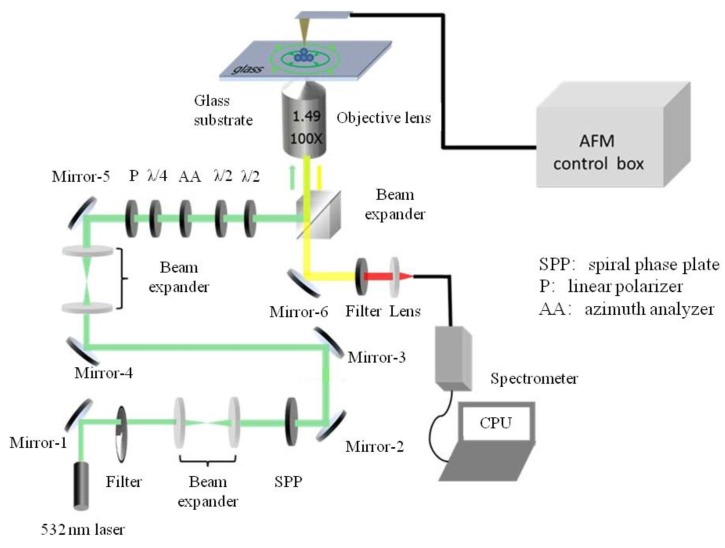
Schematic of the experiment system linked by AFM and the homebuilt confocal Raman spectrometer.

Our experimental system was partly homebuilt which was a linkage system with an AFM (NT-MDT Spectrum Instruments, Moscow, Russia) and a confocal Raman microscope equipped with a nitrogen cooled spectrometer (Horiba iHR550). As shown in Figure 9, the confocal Raman microscope worked in the inverted mode thus could be easily incorporated with the AFM. The optical path started with a 532 nm continue laser (Laser Quantum Inc.), and then consisted of a beam expander, a spiral phase plate (SPP) (Holo/Or Ltd., Israel), another beam expander, and then a set of optical plates including a polarizer, a quarter-wave plate, an azimuth analyzer (TSI Model 10901A, Thermo Oriel, Stratford, CT, United States), and two half-wave plates. This part of the optical path could generate the radially polarized optical beam, which was then focused by an oil immersion objective (Olympus, 100× with NA of 1.49). At the Raman signal collection path, there was an edge filter (Semrock) and an optical fiber with diameter of 100 μm (Thorlabs) which worked as a confocal pinhole. 

### 4.2. Sample Preparation

To produce the Au@Ag-4MBA@PVP nanoparticles used in our experiments, the first step was to produce Au nanoparticles with size about 35 nm. This was done by heating the 200 mL chloroauric acid of 0.1 g/L (Shanghai Shenbo Chemical Co., Ltd., Shanghai, China) to boiling, quickly adding 2 mL 1% sodium citrate (Shanghai Heiwei Co., Ltd., Shanghai, China), and boiled for 30 minutes. The solution changed to red which indicated the formation of Au nanoparticles. The second step was to grow an Ag layer about 15 nm in thickness: the solution of Au nanoparticles were hearted to boiling, 16 mL 1% trisodium citrate solution added, and then 12 mL 10 nM silver nitrate slowly dripped (both from Shanghai Shenbo Chemical Co., Ltd., Shanghai, China) and heated for 1 h. The color of the solution changed to dark brown. As the third step, 50 mL of prepared Au@Ag nanoparticle solution was taken, 50 mL of 10 mM 4MBA Raman molecules added (Shanghai Darui Chemical Co., Ltd., Shanghai, China), and stirred overnight. As the final step, a PVP layer was encapsulated by suspending the Au@Ag–4MBA nanoparticles into 10 mg/mL PVP solution (Sinopharm Chemical Reagent Co., Ltd., Shanghai, China) and stirred for 12 h, and the solution was centrifuged and the nanoparticles were suspended in water.

To prepare the sample on a microslide, the nanoparticle solution was diluted and dripped onto a clean microslide, and then heated with a hot plate at 65 °C for 10 minutes to dry. 

**Figure 10:**
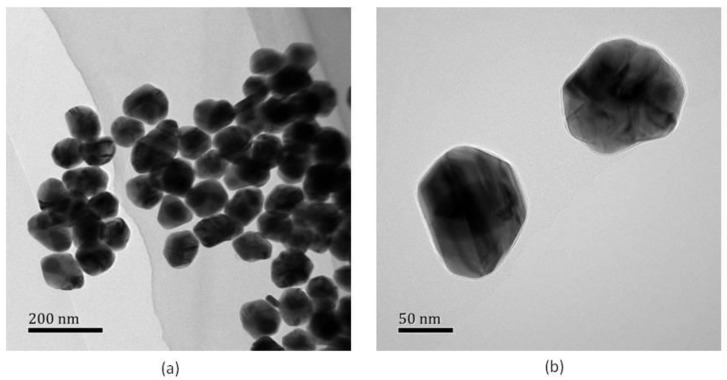
TEM graphs of the multilayer plasmonic nanoparticles at two different magnifications.

As shown in the TEM graphs (with instrument of JEM-1200EX, JEOL Ltd., Tokyo, Japan) of Figure 10, although the nanoparticles are not perfectly rounded in shape, their sizes are mostly around 100 nm. Moreover, the outer thin layer of PVP could be clearly visualized in the higher magnification graph (Figure 10b).
